# B-Lymphoid Blast Phase–Chronic Myeloid Leukemia: Current Therapeutics

**DOI:** 10.3390/ijms231911836

**Published:** 2022-10-05

**Authors:** Binoy Yohannan, Binsah George

**Affiliations:** Department of Hematology-Oncology, The University of Texas Health Science Center at Houston, 6431 Fannin Street, Huston, TX 77030, USA

**Keywords:** chronic myeloid leukemia, lymphoid blast crisis, Philadelphia chromosome, tyrosine kinase inhibitors

## Abstract

Blast crisis (BC) is one of the most dreaded complications of chronic myeloid leukemia (CML). Fortunately, the incidence of BC has diminished markedly in the *BCR-ABL* tyrosine kinase inhibitor (TKI) era. The primary objective of initial treatment in BC is to achieve a second chronic phase (CP) and to proceed to an allogeneic stem cell transplantation (SCT) in eligible patients. The clinical outcome of patients with CML BC remains unsatisfactory, even with highly potent TKIs, as remissions are short lived and there is an unmet need for novel therapies. We provide a comprehensive summary reviewing the current management of Lymphoid BC.

## 1. Introduction

**Figure 1:**
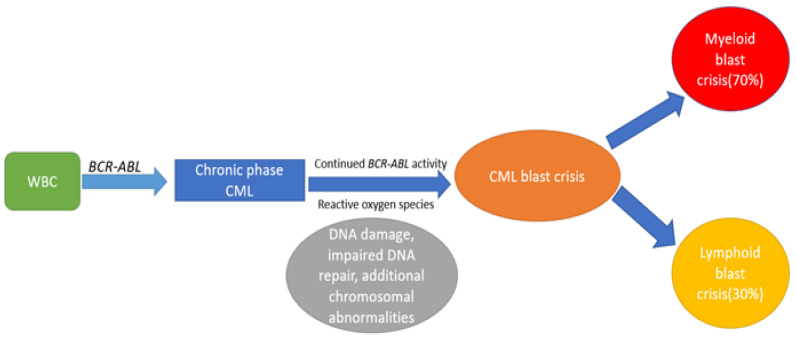
Shows the mechanisms involved in progression from CP to BC.

Chronic myeloid leukemia (CML) is a triphasic myeloproliferative disorder that accounts for 15–20% of adult leukemias [1]. The cytogenetic hallmark is reciprocal translocation between chromosome 9 and 22, resulting in *BCR-ABL 1* fusion gene, also known as the Philadelphia chromosome. As CML evolves from CP to BC, it acquires additional chromosomal anomalies beyond the Philadelphia chromosome. The majority of CML cases (>90%) are diagnosed in the CP, whereas a minority (2.2%) may present with *de novo* blast crisis [2]. BC arises due to continued *BCR-ABL* activity, leading to genomic instability and accumulation of additional chromosomal abnormalities. The natural progression from CP to BC and the underlying mechanisms are shown in Figure 1. 

In the absence of treatment, almost all patients with CP-CML will progress to BC in 3–5 years, but this dreaded transformation has become a rare event in the TKI era. Long-term outcomes from the IRIS study (interferon vs. imatinib) have shown that the risk of blast transformation is around 6.9% over 10 years [3].

Patients with blast transformation may present with symptoms of acute leukemia (e.g., bleeding diathesis, bone pain, night sweats, weight loss, fatigue). Initial evaluation of patients with BC should include complete blood counts, comprehensive metabolic panel, bone marrow aspiration, and biopsy. The latter should be sent for flow cytometry, immunohistochemistry, and cytogenetics. As per the World Health Organization (WHO) definition, BC is defined as ≥20% blasts in the bone marrow or peripheral blood. Evidence of extramedullary disease or myeloid sarcoma is also diagnostic. The majority of CML BC cases belong to the myeloid lineage, but up to one-third may transform to lymphoid BC^2^. This review summarizes the current treatment approach and emerging therapies in Lymphoid BC.

Lymphoid blast crisis (BC) accounts for almost 30% of CML BC, with the B-cell lineage being more common [4]. Very rarely, patients can present with T-cell BC [5]. Lineage switch is a rare event in which patients start with myeloid BC and switch to lymphoid BC or vice versa [6]. Lymphoid BC in CML has distinct clinical and hematological features. Clinical highlights include less frequent hepatomegaly and splenomegaly when compared to non-lymphoid patients. Additionally, patients with lymphoid BC tend to have a lower blood basophil count and a higher bone marrow blast percentage. Interestingly, lymphoid BC patients may develop *de novo* BC, without the intervening accelerated phase (AP), more often than non-lymphoid patients [7].

It is often challenging to differentiate CML lymphoid BC from de novo Philadelphia positive (Ph+) acute lymphoblastic leukemia (ALL), especially if the morphologic features of CML are absent. However, it is critical to make the distinction between these two entities, as it has important therapeutic implications. Pre-B-cell is the cell of origin in Ph(+) B-cell ALL, whereas granulocyte-macrophage progenitor is the cell of origin in blast phase CML [8]. FISH studies can detect the isoforms of *BCR-ABL* oncogene and provide important clues. The p210 isoform is commonly seen in CML, whereas the p190 isoform occurs in the majority of Ph + ALL [9]. Additionally, dipeptidyl peptidase-IV (DPP IV/CD26) is a cytokine-targeting surface enzyme which is considered a novel surface marker of CML Leukemic Stem Cell (LSC) [10]. Flow cytometry can identify this novel surface marker in peripheral blood, and this can be used as a helpful tool for CML diagnosis [11].

### 1.1. Management of CML Lymphoid Blast Crisis

The primary objective in the management of BC is to achieve a second CP and proceed to an allogenic SCT in eligible patients. At the beginning of the early 1970s, patients were treated with acute leukemia chemotherapy protocols, and it was noted that up to one-third of the patients had a brief response to a combination of vincristine and prednisone [12]. The overall response rates to induction chemotherapy are around 30%, but these responses are often transient, and the median survival was only 6–8 months [13,14]. Building on that platform, newer antileukemic chemotherapy protocols have been combined with TKI to achieve a better response.

### 1.2. Role of TKIs in Lymphoid Blast Crisis

CML lymphoid BC is often treated with chemotherapy protocols for Ph+ ALL in combination with a TKI. Single agent imatinib has limited activity in BC with a complete cytogenetic response (CCyR) of around 10% and median overall survival (OS) of 7 months. Patients who progressed to BC while on a TKI should have a *BCR-ABL1* mutation analysis to guide appropriate TKI selection. A second- or third-generation TKI is preferred in CML patients who progressed to lymphoid BC on imatinib. Dasatinib has the added advantage that it can cross the blood–brain barrier in a more efficient manner. Thus, it would be a good therapeutic option for patients presenting with extramedullary blast crisis involving the central nervous system [15]. A third-generation TKI such as Ponatinib has exhibited significant clinical activity in the phase II PACE trial, achieving a MCyR of 23% in patients with BC. Clinical activity of single agent first-, second-, and third-generation TKIs in patients with CML Lymphoid BC is summarized in Table 1. All the TKIs except nilotinib are FDA approved in BC.

### 1.3. TKI Plus Chemotherapy in CML Lymphoid Blast Crisis

High-intensity chemotherapy regimens can usually achieve excellent molecular response; however, they are associated with high induction mortality and morbidity. Hence, they are usually reserved for relatively young patients with excellent performance status. Strati et al. reported outcomes of 42 patients with CML lymphoid BC treated with imatinib or dasatinib in combination with hyper CVAD (hyper-fractionated cyclophosphamide, vincristine, Adriamycin, and dexamethasone). In total, 90% of patients achieved a CHR, 58% had CCyR, and 42% had no evidence of minimal residual disease. Median OS was 17 months and longer in patients those who proceeded to an allogenic SCT [25]. In another phase II study of 34 patients with relapsed/refractory (R/R) Ph+ ALL or CML Lymphoid BC with prior TKI therapy (*n* = 15 with lymphoid BC), the combination of dasatinib and hyper CVAD yielded an impressive objective response rate (ORR) and complete response (CR) rate was 91% and 71%, respectively. The 3-year OS for patients with lymphoid BC was 70% [26]. Similarly, Ponatinib combined with hyper CVAD has shown outstanding results in patients with Ph+ ALL with CR of 100%, event-free survival of 70% at 3 years and can be extrapolated to lymphoid BC [27].

Morita et al., in a retrospective study, compared clinical outcomes of patients with CML lymphoid BC (*n* − 19) with Ph+ ALL (*n* = 62) who were treated with Hyper CVAD plus dasatinib therapy. Morphological response rates (CML-LBC—90% vs. Ph+ ALL—96%) and CCyR (94% with CML-LBC vs. 95% Ph+ ALL) were similar. Major molecular response (MMR) was achieved in 68% with CML-LBC vs. 95% with Ph-positive ALL. Complete molecular response (CMR) was achieved in 53% with CML-LBC vs. 74% with Ph-positive ALL [28]. For ineligible patients, or those without suitable donors for an allogenic SCT, a second- or third-generation TKI with hyper CVAD would be an excellent option to attain long-term leukemia-free survival.

A lower-intensity regimen is often better tolerated in elderly or frail patients. Rea et al. investigated the DIV regimen consisting of dexamethasone, imatinib (800 mg daily), and vincristine in patients CML Lymphoid BC (*n* = 13). After induction, 11/13 with lymphoid BC (84%) achieved CHR and rates of MCyR and CCyR were 46% and 31%, respectively [29]. A brief summary of the studies are provided in Table 2. 

In the EWALL-PH-01 international study, patients with Ph+ ALL were treated with a combination of dasatinib, vincristine, and dexamethasone (DVD). Elderly patients with a high comorbidity score were able to tolerate this regimen with a CR rate of 95%- and 5-year OS of 36% [30]. Given the excellent response seen with the DIV regimen in lymphoid BC, DVD would offer a very promising low-intensity regimen incorporating a second-generation TKI that could yield better results. A chemotherapy-free regimen consisting of dasatinib and dexamethasone can achieve high CR rates (97%) with minimal risk of induction mortality, and has been used successfully in Ph+ ALL [31]. This regimen appears promising and needs to be investigated further in CML lymphoid BC. For patients with comorbidities that preclude the use of anthracyclines, a chemoimmunotherapy regimen consisting of mini hyper CVD (50% dose reduction for cyclophosphamide and dexamethasone and omitting anthracycline) in combination with inotuzumab has shown impressive results in Philadelphia negative R/R ALL [32]. Updated results from a phase II study of mini hyper CVD plus inotuzumab, with or without blinatumomab, in older adults with newly diagnosed Philadelphia negative ALL has shown outstanding results with an overall response rate of 99%- and 5-year OS of 47% [33]. The addition of a second- or third-generation TKI to this regimen could be an exciting option that needs to be explored further in CML lymphoid BC. Given the risk of central nervous system (CNS) relapse, patients with lymphoid BC should receive prophylactic intrathecal chemotherapy (ITC) with either methotrexate or cytarabine. Patient should receive a minimum of eight ITC treatments, as studies have shown that patients with Ph+ ALL who receive less than eight ITC treatments have a significantly higher risk of CNS relapse [34,35].

### 1.4. Emerging Therapies

(A) Monoclonal antibodies, bispecific antibody, and novel TKIs:
  1. Inotozumab ozygomycin (IO), monoclonal antibody targeting CD-22 in combination with bosutinib has shown robust clinical activity. In a phase 1/2 study of 18 patients (*n* = 2 with lymphoid BC), the combination of IO and bosutinib achieved an ORR of 83% and CMR of 56% [36]. For patients with R/R CML lymphoid BC, single-agent Inotuzumab ozogamicin has been used successfully as salvage therapy, even in patients with blinatumomab failure [37]
  2. Blinatumomab, a bispecific T-cell engaging CD3–CD19 antibody, in combination with TKI has excellent activity in Ph+ leukemia and has been shown to be safe and effective, resulting in deep and durable remissions [38]. Dasatinib in combination with blinatumomab has shown impressive results in Ph+ ALL [39] and could be an effective option with low-induction mortality in lymphoid BC. In a retrospective study of 18 patients with R/R Ph+ leukemias (*n* = 2 with lymphoid BC) treated with blinatumomab and TKI, both patients achieved an MRD negative remission and were able to proceed to allogenic SCT [40]. Blinatumomab in combination with ponatinib was investigated in a single-arm phase 2 study of 35 patients with newly diagnosed (*n* = 20), (R/R) Ph+ ALL (*n* = 10) and CML lymphoid BC (*n* = 5), achieving an impressive ORR of 100% in newly diagnosed Ph+ ALL, 88% in R/R group. Among responders, 86% achieved CMR (87% in the newly diagnosed group and 86% in the R/R group) and 40% in CML lymphoid BC. Two-year OS in the newly diagnosed cohort, R/R group, and CML cohort was 93%, 53%, and 100%, respectively [41]. Hence, blinatumomab in combination with a third-generation TKI appears to be a highly effective regimen that is well tolerated.
  3. A chemotherapy-free regimen consisting of venetoclax, dexamethasone, and ponatinib has impressive activity in patients with R/R Ph+ ALL. In a phase I/II study of R/R Ph+ ALL and CML Lymphoid BC, a combination of venetoclax, dexamethasone, and ponatinib yielded a 50% CR. All patients who received 800 mg of venetoclax achieved a CMR [42]. This study is still ongoing and appears promising.
  4. Asciminib is a novel allosteric inhibitor of *BCR-ABL* fusion protein that is approved in patients with CP-CML those who have been previously treated with two or more TKIs [43]. Additionally, preclinical studies have shown that there is synergistic activity when combining Asciminib and ponatinib [44]
  5. Olverembatinib is a third-generation *BCR-ABL* TKI which is highly effective in CML patients with T315I mutation. In a phase II study (CC 202) of 23 patients with CML-AP, Olverembatinib achieved a CHR of 60.9% and CCyR of 39.1% [45].
  6. PF114 is an oral TKI similar to ponatinib and was studied in a phase I/II study of 51 patients with CP/AP CML resistant to two or more TKIs. In the cohort treated with 300 mg daily, the rates of MCyR and MMR were 54% and 33%, respectively [46].
  7. CD 123 (Interleukin-3 receptor) is highly expressed on CML stem cells, and the level of expression correlates with disease progression. CD123 targeting antibodies are already in clinical use for treating blastic plasmacytoid dendritic cell neoplasm (BPDCN) [47]. Preclinical studies have shown that antibodies targeting CD 123 are highly effective at killing blasts and eradicating LSC [48]. Clinical activity of CD 123 antibodies in combination with TKIs also needs to be explored further.
  8. Interleukin-2 in conjunction with CD25 expressed on CML cells constitutes a unique leukemia-initiating cell niche. In mice model, it has been shown that blocking the IL-2/CD25 receptor can eliminate the leukemia initiating cell and improve survival [49].
  9. Interferon alpha (IFN-α) either as single agent or combined with TKI may offer a novel strategy to counter TKI-resistant clones. Ilander et al. reported a patient who was treated with imatinib for 35 months and developed hematological relapse secondary to T315I mutation. Ten weeks of IFN-α resulted in deep molecular response [50]. Polivkova et al. reported a series of six cases treated with IFN-α based therapy, resulting in a reduction of T315I or compound mutations to undetectable and 4/6 patients achieving a molecular response [51].
(B) Immunotherapy and cellular therapy:
  1. Chimeric Antigen Receptor (CAR) T cell therapy is an exciting novel cellular therapy option to eliminate the CML stem cells, and this modality can be helpful for TKI-resistant/intolerant patients with advanced phase CML. Additionally, CD 19-directed CAR-T cell therapy could prove to be an effective therapy in RUNX1 mutant CML BC with aberrant expression of CD19 cells. Ex vivo studies have shown excellent cytotoxic activity of CD-19 CAR T cells in both myeloid and lymphoid CML BC [52]. Imatinib in combination with CD19-CAR T cells was highly active in the killing of *RUNX1* mutant CML BC cells and the latter was able to successfully kill imatinib-resistant clones. Allogenic CAR T cells appears to be an exciting new therapeutic option in patients with refractory CML lymphoid BC that has failed a stem cell transplant [53]. Additionally, in patients with mixed-phenotype CML BC, which poses a therapeutic challenge, CD 19 CAR T cell therapies have been used successfully to achieve durable remissions [54]. CAR T cell therapy appears to be active in T315I mutant BC. Zhou et al. published an interesting case of a patient with T315I mutant CML lymphoid BC who was treated with CD19 CAR-T therapy and dasatinib achieving a complete molecular remission. It can be hypothesized that CD19 CAR-T cells eliminated the T315I mutant clone, which allowed dasatinib to work effectively [55]. Another target would be Interleukin receptor 1 receptor accessory protein (IL1RAP), expressed on CML cells [56]. Preclinical studies have shown that anti-IL1RAP-CAR-T therapy can effectively eliminate CML stem cells without major off-target toxicity [57].
  2. Patients with *RUNX1* mutations have a distinct phenotypic picture characterized by aberrant expression of lymphoid antigens (CD19, CD7), and this provides several exciting molecular targets for CART-T cell immunotherapy [52]
  3. NK Cell therapy: Kim et al. reported that in patients with advanced, multi-TKI-resistant CML, donor natural killer (NK) cells can effectively kill CML blasts irrespective of BCR-ABL1 mutations. NK cells can also eliminate leukemic stem cells (LSC) [58]. This is an exciting new concept and a viable strategy that needs to be explored further.

### 1.5. Role of Allogenic SCT in CML BC

Allogeneic SCT is an important pillar in CML therapy and the only curative option. It offers the best chance of long-term leukemia-free survival (LFS). Pre-transplant remission status is another important factor determining long-term clinical outcomes in patients undergoing allogenic SCT for BC. The French CML group looked at clinical outcomes of 63 patients CML BC, and it was observed that the disease status at time of allografting and the EBMT score were the most important prognostic factors [59].

Radujkovic et al. reported a retrospective review of 170 adults who underwent allogenic SCT for CML BC. Estimated 3-year OS for patients with active disease at the time of SCT was 23.8% compared to 51.1% for those in remission. Similarly, the 3-year LFS in patients who were allografted in remission was three-fold higher compared to those with active disease (11.6% vs. 33.8%) [60]. The type of BC (myeloid vs. lymphoid) had no impact on post-transplant LFS or OS. A CIBMTR (Center for International Blood and Marrow Transplant Research) study of 449 patients (80 with BC) also confirmed the same finding that patients going into a SCT with active disease had significantly worse outcomes, and in this group, using an unrelated donor was associated with better leukemia-free survival [59,60]. The role of post SCT TKI maintenance is controversial. Although TKI maintenance for 12 months is recommended by the NCCN, data from the CIBMTR study showed no benefit in terms of LFS and OS [61].

### 1.6. Prognosis

The most important determinant of prognosis in patients with CML BC is the response to therapy [62,63]. Patients those who were able to achieve a MHR and/or CCyR to first-line therapy had significantly better survival [64].

In a large retrospective study from MDACC of 477 patients with CML BC, Jain et al. reported better overall survival (OS) for patients with de novo BC compared to those with transformed CML-CP/CML-AP. The cell lineage of the blast and prior TKI treatment also had an impact on survival. Patients with lymphoid BC and those with no TKI exposure had better survival compared to those with myeloid phenotype and prior TKI therapy before blastic transformation. Older age (≥58 years), high LDH l (≥1227 IU/L), thrombocytopenia (<102 × 10^9^/L) and chromosome 15 alteration were considered adverse prognostic factors [64].

## 2. Conclusions

**Figure 2:**
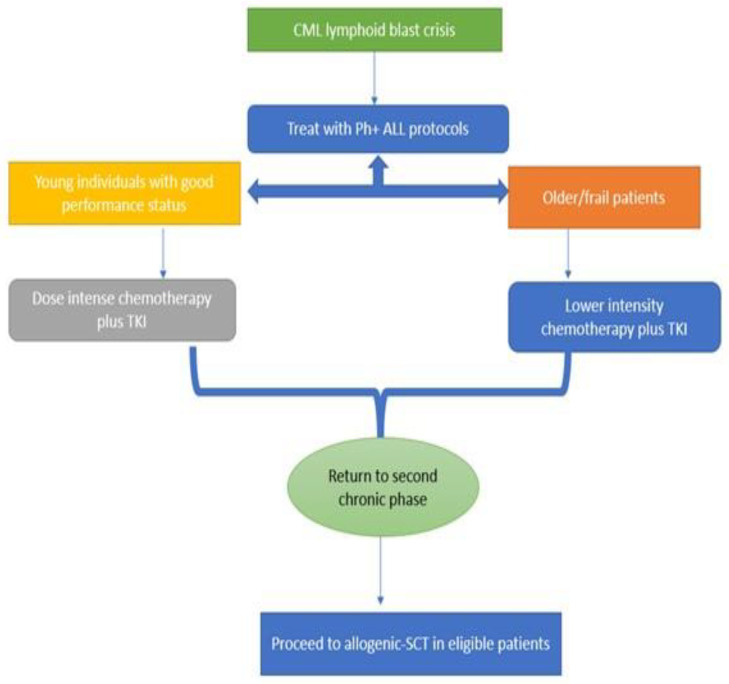
Flow chart showing therapeutic approach in CML lymphoid BC.

The algorithm in Figure 2 gives a broad overview of the management of CML lymphoid BC. Although the incidence of BC has diminished significantly in the TKI era, clinical outcomes remain suboptimal. Additionally, given the rarity of the disease, there is a paucity of high-quality data to guide treatment decision. Prevention of progression to BC is the most effective strategy, and this can be achieved by adapting evidence-based management of CP CML. Clinical trial participation should be highly encouraged in eligible patients.

## Figures and Tables

**Table 1:** Various TKIs in patients with Lymphoid Blast Crisis.

TKI	Study	Total Patients (*n*)	Dose (mg)	Lymphoid Blast Crisis					
				*n*	HR (%)	CHR (%)	CyR		Median Survival (Months)
							MCyR (%)	CCyR (%)	
Imatinib	Druker et al. [16]	58	300–1000	20	70	20	15	10	na
	Kantarjian et al. [17]	75	300–1000	10	30	10		10	6.8
	Palandri et al. [18]	92	600	20	60	35		20	7
Nilotanib	Giles et al. [19]	136	800	31	59	21	52	32	7.9
	Kantarjian et al.	119	50–1200	9	33	0	11	11	na
	Nicolini et al. [20]	190	800	50	28	14	36	26	66% at 12 m
Dasatinib	Cortes et al. [21] (Imatinib R/I)	116	140	42	36	26	50	43	26% at 24 m
	Talpaz et al. [22] (Imatinib R/I)	84	15–240	10	80	70	80	30	6
	Saglio et al. [23]	214	140	61	38	21	46	38	11.4
Ponatanib [24]	Cortes et al. [24]	62	45	10	40	na	40	30	29% at 12 m

HR—Hematological Response, CHR—Complete Hematological Remission, CyR—Cytogenetic Remission, MCyR—Major Cytogenetic Remission, CCyR—Complete Cytogenetic Remission. R/I—Resistant or intolerant.

**Table 2:** Summary of chemotherapy plus TKI in Lymphoid Blast Crisis.

TKI+ Chemotherapy Regimen	Study	Total Patients (*n*)	CHR (%)	CCyR (%)	CMR (%)	Median OS in Months
Imatinib or dasatinib combined with Hyper CVAD	Strati et al. [25]	42	90	58	25	17
Dexamethasone + imatinib + vincristine (DIV Regimen)	Rea et al. [29]	13	84	31	11	13.5
Dasatinib plus Hyper CVAD	Benjamini et al. [26]	15	7	71	36	70% at 36 m

HyperCVAD—hyperfractionated cyclophosphamide, vincristine, Adriamycin, dexamethasone; CHR—Complete Hematological Response; CCyR—Complete Cytogenetic Response; CMR—Complete Molecular Response.
